# Blame-rebalance fMRI neurofeedback in major depressive disorder: A randomised proof-of-concept trial

**DOI:** 10.1016/j.nicl.2019.101992

**Published:** 2019-08-25

**Authors:** Roland Zahn, Julie H. Weingartner, Rodrigo Basilio, Patricia Bado, Paulo Mattos, João R. Sato, Ricardo de Oliveira-Souza, Leo F. Fontenelle, Allan H. Young, Jorge Moll

**Affiliations:** a Cognitive and Behavioral Neuroscience Unit, Neuroinformatics Workgroup, D'Or Institute for Research and Education (IDOR), Rio de Janeiro, Brazil; b Instituto de Ciências Biomédicas (ICB), Universidade Federal do Rio de Janeiro, Rio de Janeiro, Brazil; c Center for Mathematics, Computation, and Cognition, Universidade Federal do ABC, Santo André, Brazil; d Gaffrée e Guinle University Hospital, Federal University of the State of Rio de Janeiro, Rio de Janeiro, Brazil; e Centre for Affective Disorders, Institute of Psychiatry, Psychology & Neuroscience, King's College London, United Kingdom; f Scients Institute, Palo Alto, USA

**Keywords:** Real-time fMRI, fMRI neurofeedback, Clinical trial, Guilt, Major depressive disorder, Anger, Subgenual cingulate cortex, Anterior temporal lobe, Self-esteem

## Abstract

Previously, using fMRI, we demonstrated lower connectivity between right anterior superior temporal (ATL) and anterior subgenual cingulate (SCC) regions while patients with major depressive disorder (MDD) experience guilt. This neural signature was detected despite symptomatic remission which suggested a putative role in vulnerability. This randomised controlled double-blind parallel group clinical trial investigated whether patients with MDD are able to voluntarily modulate this neural signature. To this end, we developed a fMRI neurofeedback software (FRIEND), which measures ATL-SCC coupling and displays its levels in real time. Twenty-eight patients with remitted MDD were randomised to two groups, each receiving one session of fMRI neurofeedback whilst retrieving guilt and indignation/anger-related autobiographical memories. They were instructed to feel the emotion whilst trying to increase the level of a thermometer-like display on a screen. Active intervention group: The thermometer levels increased with increasing levels of ATL-SCC correlations in the guilt condition. Control intervention group: The thermometer levels decreased when correlation levels deviated from the previous baseline level in the guilt condition, thus reinforcing stable correlations. Both groups also received feedback during the indignation condition reinforcing stable correlations. We confirmed our predictions that patients in the active intervention group were indeed able to increase levels of ATL-SCC correlations for guilt vs. indignation and their self-esteem after training compared to before training and that this differed significantly from the control intervention group. These data provide proof-of-concept for a novel treatment target for MDD patients and are in keeping with the hypothesis that ATL-SCC connectivity plays a key role in self-worth.

*https://clinicaltrials.gov/ct2/show/results/NCT01920490*

## Highlights

• Employs real-time fMRI of anterior temporal –subgenual cingulate connectivity
• Previously decreased for guilt in major depressive disorder (MDD) beyond remission
• This RCT shows MDD patients can increase connectivity in one neurofeedback session.
• Active neurofeedback group increase self-esteem vs control neurofeedback group
• Training-induced self-esteem increases correlate with connectivity increases

## 1 Introduction

FMRI neurofeedback provides individuals access to information about their current local brain activity that is usually outside of their awareness. This potentially mitigates a shortcoming of cognitive therapy (Beck et al., 1979) for major depressive disorder (MDD) which relies on information that patients are consciously aware of. FMRI neurofeedback further enables probing causal relationships between local brain function and psychological symptoms in MDD. Here, we used this approach to probe the role of functional connectivity between the right superior anterior temporal (ATL) and the subgenual cingulate cortex (SCC) in overgeneralised self-blaming emotional biases and MDD vulnerability. The influential revised learned helplessness model (Abramson et al., 1978) states that vulnerability to major depressive disorder (MDD) is due to a bias to blame oneself for failure in an overgeneralised way resulting in decreased global self-esteem and depression and this is consistent with the phenomenology and coherence of MDD symptoms (Zahn et al., 2015b) and the effectiveness of cognitive therapy which focuses on self-critical thinking (Beck et al., 1979). Overgeneralised self-blame is associated with excessive self-blaming emotions (Green et al., 2013) (e.g., feeling “guilty for everything” or “hating oneself in general”) and worthlessness. So far, fMRI neurofeedback interventions for MDD have been designed, however, on the basis of a model that proposes an overall increase in negative and reduction in positive emotions (Watson et al., 1988a) rather than self-blame-selective increases in negative emotions.

(Linden et al., 2012) showed in their pioneering but non-randomised trial that fMRI neurofeedback can reduce depressive symptoms in current MDD when reinforcing activation in brain regions responding to positive pictures. In a recent randomised controlled trial, they confirmed these findings, although similar levels of improvement were observed in the active neurofeedback control group (Mehler et al., 2018). (Young et al., 2014) have developed an approach based on reinforcing amygdala activation in response to positive autobiographical memories. This method has recently been probed in current MDD in a pilot double-blind randomised controlled trial (RCT), exhibiting superiority in reducing depressive symptoms versus a control neurofeedback intervention that reinforces parietal cortex activation (Young et al., 2017). Another study showed a decrease in negative emotions on decreasing activation in salience-related brain regions including the amygdala in response to negative pictures in depression (Hamilton et al., 2016).

We recently provided evidence that reduced positive memory biases, as successfully targeted in previous neurofeedback studies (Young et al., 2017; 2014), were only present in a subgroup of people with MDD. It was only those patients with early life stress who had reduced positive memory biases and these were associated with number of previous episodes, suggesting their role in “scarring” which may increase future vulnerability (Gethin et al., 2017). This indicates that neurofeedback interventions targeting positive memories may be most effective in MDD patients with early life stress. So far, however, fMRI neurofeedback interventions aimed at self-blaming emotional biases in MDD, shown to be independent from general negative emotional biases (Green et al., 2013; Zahn et al., 2015a), are lacking.

In this double-blind RCT we sought to establish proof-of-concept for a neurofeedback approach aimed at self-blame-selective reductions in functional connectivity (Sato et al., 2013) between the right superior anterior temporal (ATL) and subgenual cingulate cortex (SCC), previously identified as a signature of overgeneralised self-blaming emotions in MDD (Green et al., 2012). This signature was found in patients with remitted MDD (Green et al., 2012), known to have an increased vulnerability towards MDD (Eaton et al., 2008). Functional disconnection of the ATL and SCC was found while people felt guilty during fMRI relative to feeling indignation. We sought to determine whether self-blame-selective disconnection on fMRI can be detected and fed back to the participants after a short temporal delay with real-time fMRI and whether connectivity can be increased through neurofeedback training in people with remitted MDD. This work builds on extensive evidence for the pathophysiological importance of SCC networks in MDD (Drevets and Savitz, 2008; Dunlop et al., 2017; Price and Drevets, 2010; Ressler and Mayberg, 2007; Siegle et al., 2006). Our pre-registered specific aims were (ClinicalTrials.gov: NCT01920490):

1) Demonstrate that ATL-SCC coupling for guilt can be increased through one session of neurofeedback in the group seeing visual feedback based on increasing correlations during the guilt condition compared with the group seeing visual feedback based on keeping correlations at the same level during the guilt condition.
2) Demonstrate that this increase in coupling is selective for guilt relative to indignation.
3) Demonstrate that mood is not negatively affected by neurofeedback.
4) Explore whether this short intervention decreases self-hate on the Interpersonal Guilt Questionnaire (O'Connor et al., 1997) and increases self-esteem on the Rosenberg global self-esteem scale (Rosenberg, 1989) (both showed significant correlations with SCC-ATL coupling across major depressive disorder and control groups in our previous study (Green et al., 2012)), or decreases negative affect on the Positive and Negative Affect Scale (PANAS (Watson et al., 1988b)).

## 2 Materials and methods

This randomised, controlled, double-blind, parallel group, clinical trial was pre-registered (*https://clinicaltrials.gov/ct2/show/results/NCT01920490*).

### 2.1 Participants

*Inclusion criteria:* past major depressive episode according to the Diagnostic Statistical Manual (DSM-IV) for at least 2 months, currently not fulfilling criteria for an episode and remitted from symptoms for at least 2 months, and age ≥18.

*Exclusion criteria*: current suicidal thoughts*,* other current DSM-IV axis-I disorders*,* a history of atypical major depressive episodes (DSM-IV)*,* Global Assessment of Functioning scores below 80 as a sign of incomplete remission or co-morbidity*,* >2 points on the suicidality item of the Hamilton Depression Scale (Hamilton, 1960)*,* prior criminal convictions*,* history of violent behaviour towards persons as determined by clinical interview*,* positive past or current screening question for irritability on the mood disorders module*,* antisocial or borderline personality disorder as determined on a personality interview using DSM-IV criteria*,* or current self-harming behaviours.

Twenty-eight patients (Table 1) completed the study out of 32 patients enrolled between May 2013 and October 2014 at the D'OR Institute for Research and Education, Rio de Janeiro, Brazil. We did not employ a formal power calculation to determine the required sample size due to a lack of useful effect size estimates, as no comparable previous study had been carried out. One patient was excluded prior to randomisation due to a diagnosis of a borderline personality disorder on detailed assessment. Of the 31 patients allocated to the intervention, 3 had to be excluded due to technical problems with MRI scanning (for CONSORT Flow Diagram, see Supplementary Fig. 2). All participants were native Brazilian-Portuguese speakers with normal or corrected-to-normal vision.

**Table 1|:** Clinical and demographic characteristics of the intervention groups.

	Active (n = 14)	Control (n = 14)
Number of previous MDEs (percentiles)	25th = 1 50th = 2, 75th = 3	25th = 1 50th = 2, 75th = 10
	(range: 1–4)	(range: 1–20)
Psychotic symptoms in previous MDE	0	2
Current medication		
SSRI	6	8
SNRI	3	1
Tricyclic antidepressant (therapeutic dose)	1	1
Low dose tricyclic antidepressant add-on	1	0
Topiramate	1	1
No antidepressant medication	4	4
Benzodiazepines	7	7
Ritalin	1	0
Life-time co-morbidity		
Bulimia nervosa	1	0
Anorexia nervosa	0	1
Panic disorder/agoraphobia	3	1
Social phobia	2	0
Obsessive Compulsive Disorder	0	1
Generalised Anxiety Disorder	1	0
Specific phobia	1	3
Health anxiety disorder	0	1
Multiple anxiety disorders	1	3
No anxiety disorder	6	5
Substance abuse	0	1
Alcohol abuse	2	1
Alcohol and substance abuse	1	0
No substance or alcohol abuse	11	12

Participants in the Active and Control Intervention groups did not differ on median number of previous episodes despite higher 75th percentiles in the CONTROL group. Number of cases are reported. MDE, major depressive episode; SSRI, selective serotonin reuptake inhibitor; SNRI, serotonin norepinephrine reuptake inhibitor. One participant in the CONTROL group (SUBJ0003) showed bipolar features on the MINI interview which were not deemed to meet criteria for bipolar disorder by the senior psychiatrist. Interestingly, this participant showed the highest connectivity for guilt relative to indignation at baseline of the whole study, yet the significant group differences in baseline connectivity for guilt vs. indignation remain even when excluding SUBJ0003 (*t* = 2.1, *p* = .05).

Participants were randomly allocated by the FRIEND software using a text file (Basilio et al., 2015; Sato et al., 2013) into the two intervention groups (*n* = 14 in each group, Active: 4 male, Control: 3 male) without researchers being aware of the allocation. After seeking statistical advice, we amended our protocol to use minimised randomisation (Roberts and Torgerson, 1998) to avoid group imbalances in demographic confounders rather than a simple randomisation algorithm with no stratification (Sato et al., 2013). Minimisation was implemented by first using random assignment to the two groups and then assigning further patients to the group which minimizes group differences in running totals for mean age and gender distribution. Minimisation was carried out by Sebastian Hoefle, an IDOR employee who was not part of the research team and had no access to participant data apart from participant ID, age and gender information. After carrying out the minimised random allocation, he saved the allocation in a text file which he uploaded onto the FRIEND server directly, thereby assuring concealment from the research team. Researchers were unblinded only after completing all assessments by looking at the text file that indicated the group allocation. Participants were not unblinded. Written informed consent was obtained from all participants. The study was approved by the Ethics and Scientific committees of the Federal University of Rio de Janeiro (Study number 05089412.2.1001.5263) and the D'Or Institute for Research and Education (Study number 05089412.2.2002.5249).

### 2.2 Clinical assessment

Patients were referred by their clinical psychiatrists and neurologists (P.M., L.F., R.O.) affiliated with IDOR, and screening assessments were carried out by an independent clinical researcher as part of the IDOR neuropsychiatric and normal control studies recruitment procedures. During the screening visit, a closely supervised specialist trainee and/or neuropsychologist carried out an in-depth assessment using the MINI plus International Neuropsychiatric Interview (Lecrubier et al., 1997) changing the assessment to life-time (“have you ever”) for DSM-IV, which was modified to allow for subtypes of depression to be assessed for past episodes (melancholic, atypical) and the Global Assessment of Functioning (GAF) Scale. A Hamilton Depression Scale and a personality interview using DSM-IV criteria comprising the main domains of daily functioning were carried out and all assessments were supervised by a senior psychiatrist (P.M.), who was also responsible for managing suicide risk.

### 2.3 Pre-registered outcome measures

[Time Frame for all measures: change from baseline after one session of fMRI neurofeedback training].

*Primary Outcome Measure*:

• Increase in correlation between ATL and SCC fMRI signal for guilt relative to indignation Correlations are computed by using average signal in the most highly activated voxels within a priori regions of interest (ROIs) in the right superior ATL and SCC region. The same a priori regions are also used to provide neurofeedback.

*Secondary Outcome Measures:*

• Beck Depression Inventory (BDI, (Beck et al., 1988)). This was an outcome measure to ensure the safety of our intervention, we expected that one session of fMRI neurofeedback will not lead to a significant increase in BDI scores.

The following two exploratory outcome measures were used to determine whether there is a detectable effect on self-blaming emotions after one session of fMRI neurofeedback. This was not our primary aim in that this study was primarily designed to determine feasibility and safety rather than efficacy:

• Interpersonal Guilt Questionnaire - Self-hate subscale (O'Connor et al., 1997; 2002).
• Rosenberg Self-Esteem Scale (Rosenberg, 1989)

### 2.4 FMRI paradigm design

The first and fourth run (200 volumes, 400 s duration) were identical and were used to determine pre- and post-neurofeedback effects. The first run was also necessary to define the 10% most activated voxels for guilt vs. subtraction or indignation vs. subtraction in the predefined SCC and ATL ROIs. These individualized ROIs were used to extract average signal for subsequent neurofeedback training. First and fourth runs consisted of four blocks of guilt and four blocks of indignation memories (15 volumes per block), interspersed with eight mental subtraction condition blocks (10 volumes each). The second and third run were identical, and participants were provided with visual feedback for training during these runs. The neurofeedback runs consisted of 360 volumes with 120 volumes for guilt and 120 for indignation distributed over 4 blocks (30 volumes per block) which were interspersed with 8 blocks (15 volumes) of mental subtraction (120 volumes) by serial subtraction of seven from a 3-digit number (for a graphical depiction see Supplemental Fig. 6). Mental subtraction was used to allow patients to distract themselves emotionally from the scenarios and to decrease, so-called “resting-state” activity known to be present in the SCC (Bado et al., 2014).

### 2.5 Intervention

Autobiographical events were selected by participants prior to the scanning session. They chose events that evoked guilt and entailed their own actions. They also had to choose events that evoked indignation and entailed other people acting. Participants had to define cue words pointing to these events to be displayed to them during the scanning session (2 scenarios/events for each condition). Neurofeedback instructions for both groups in both conditions were identical in that they were asked to bring up the level to which the bar is filled with colour on the thermometer-like visual feedback displayed to them whilst thinking about the event (see Supplementary Methods).

During the indignation condition, visual feedback reinforced stabilisation of the preceding degree of correlation between the ATL and SCC in both intervention groups. The two intervention groups were:

• *ACTIVE: GUILT-INCREASE-CORRELATION:* Visual feedback reinforced increasing the correlation in fMRI signal between the right superior ATL and SCC regions during retrieval of guilt-related events.
• *CONTROL: GUILT-STABILISE-CORRELATION:* Visual feedback reinforced stabilisation of the preceding degree of correlation in fMRI signal between the right superior ATL and SCC regions during the retrieval of guilt-related events. The level of the bar on the visual feedback thermometer-scale went up for correlations staying within the preceding range and down for correlations outside of the range.

The rationale for stabilisation as a control intervention was to provide feedback from the same brain regions as in the active group whilst being engaged in the same psychological task which avoids differences in the psychological aspects of the intervention in both groups. This design further avoids providing feedback from a brain region which is not relevant to the psychological aspects of the task and could thus create a mismatch between neurofeedback signal and psychological task.

### 2.6 Neurofeedback methods

**Fig. 1|:**
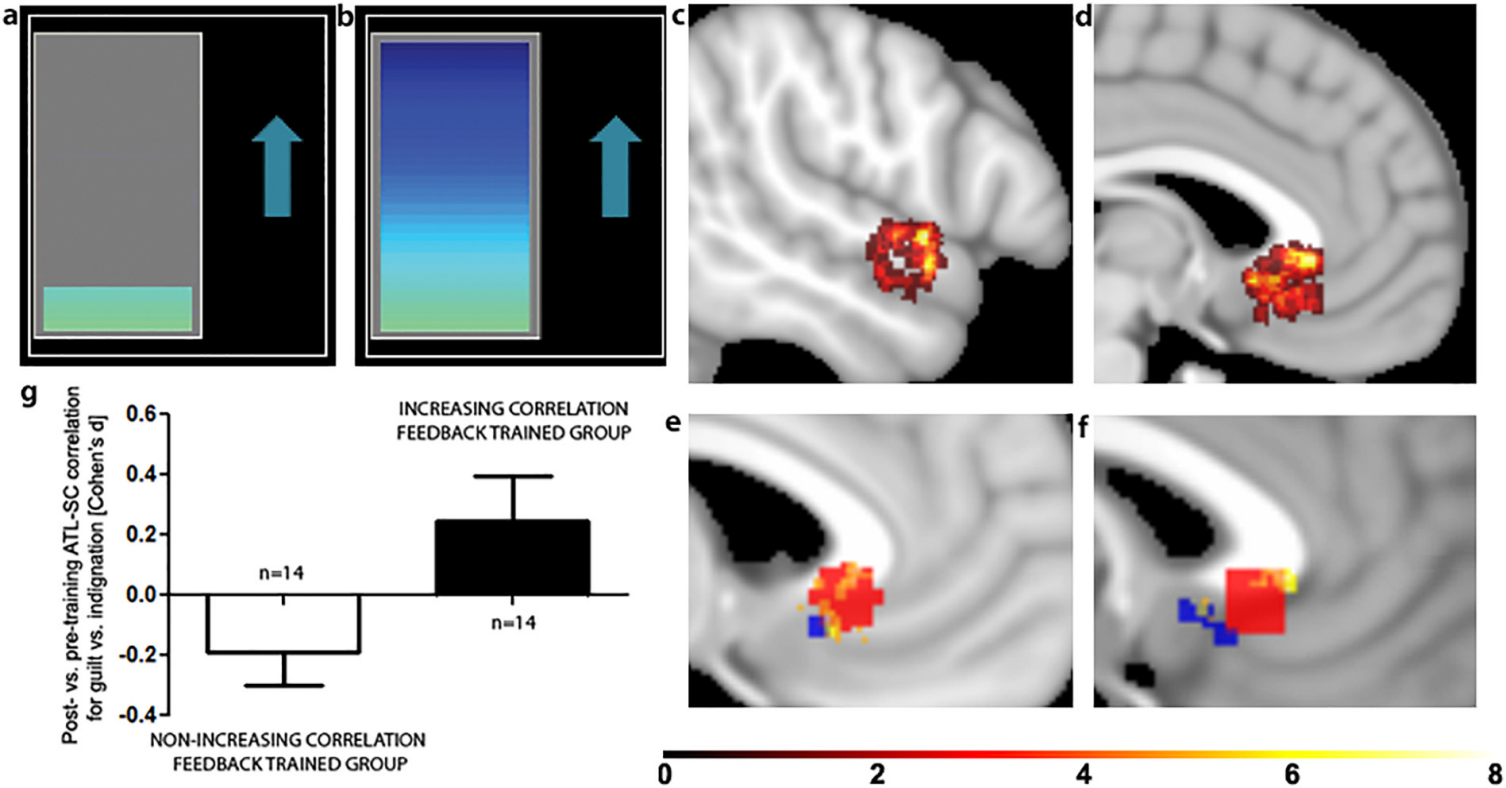
Panel a&b) Thermometer-like displays were used for visual feedback. Participants were instructed to increase the level of the colour bar (a: low level, b: high level) while thinking about the autobiographical memory related to the cue word. Panel c) & d) Displayed are the a priori anatomical regions of interest (ROI) used for extracting signal for neurofeedback training (c: ATL, d: SCC). As only the 10% most activated voxels were used for training in each subject, there was individual variability as to the sub-regions of the smoothed ROIs used for training. Colour-coded overlays indicate for how many participants a given voxel was included in their individualized ROI. Panel e) & f) Displayed are SCC voxels (Sagittal slices MNI z = −4 in e), −1 in f)) included in the training ROI for at least 4 participants overlaid with the unsmoothed anterior subgenual cingulate ROI in blue underpinning the design of this study in which we previously demonstrated self-blame-selective hypo-connectivity(Green et al., 2012) and in red the more posterior inferior subgenual cortex cluster showing self-blame-selective hyper-connectivity in remitted MDD who develop another episode in the next year in a later paper(Lythe et al., 2015). Panel g) This bar chart compares the intervention groups on neurofeedback training effects (Cohen's D for post- vs pre-training differences) in ATL-SCC connectivity for guilt vs. indignation (measured by using standardised regression coefficients). As shown in Table 2, there was a significant difference between groups in the expected direction such that connectivity for guilt vs. indignation increased with training in the active intervention group, but slightly decreased in the control intervention group. Supplementary Fig. 7 shows the individual variability in correlations during neurofeedback.

A custom-made software package (FRIEND [Functional Real-time Interactive Endogenous Neuromodulation and Decoding]; freely available at http://fsl.fmrib.ox.ac.uk/fsl/fslwiki/OtherSoftware and http://idor.org/neuroinformatics/friend (Basilio et al., 2015; Moll et al., 2014; Sato et al., 2013)) was used for real time fMRI data pre-processing including motion correction (MCFLIRT algorithm), spatial smoothing (Gaussian Kernel, Full-Width-Half-Maximum (FWHM) = 6 mm), GLM calculation, anatomically and functionally-defined ROI selection. All steps described above employed native FSL codes, within a pipeline that enabled improved processing speed (Sato et al., 2013). Signal-level normalization was performed by subtracting the mean value of the voxel signals within the ROI over the entire preceding subtraction condition block from the current echo-planar images belonging to the guilt or indignation condition block, which minimizes local signal trends. The right ATL and SCC ROI masks in MNI space were warped to subject space by using the inverse transform of the FSL-FLIRT algorithm (affine, 12-parameters). ATL (4 mm radius sphere around centre coordinate: MNI x = 58, y = 0, z = −12) and SCC ROIs (6 mm radius sphere around centre coordinate: MNI x = −4, y = 23, z = −5) were defined by using previously described a priori anatomical ROIs ((Green et al., 2012), see Supplementary Information), smoothed with 6 mm FWHM), and back-transformed into native space to then select the 10% most activated voxels in the native space ROI, this may have led to different sizes in the ROIs across subjects in native space which we were not able to control for. The activation was calculated using the contrast between guilt vs. subtraction in the ATL ROI, and guilt vs indignation in the SCC ROI. The first 5 volumes of each emotional block were discarded due to high correlations guided by decreases in time series after subtraction conditions. A moving target correlation algorithm was employed by using a sliding time window based on the last 10 volumes, updated every two seconds (i.e. for each volume). The level of the colour bar of the visual feedback signal (Fig. 1a&b) was determined on the basis of the size of the Pearson correlation coefficient measured over the last 10 volumes (weighted by a sigmoid function, see Supplementary Methods and Supplementary Fig. 1) in relation to the minimum and maximum.

Increasing correlations were reinforced by increasing the level of the colour bar if correlation coefficients were higher than the weighted mean (last 10 values) plus one standard deviation of the correlation values so far. Stabilisation of correlations was reinforced by increasing the colour bar level if correlation coefficients kept close to the weighted mean (last 10 values) and lower colour bar levels if the correlation coefficient deviated positively or negatively from the previous average.

Root mean squares (RMS) of movement parameters for translation and rotation were tracked on an ongoing basis and the neurofeedback screen displayed a warning to participants and investigators in real-time if movement exceeded allowable levels, excluding those volumes from calculations of feedback signal (see Supplementary Methods). Unfortunately, FRIEND does not record the number of these excluded volumes during real-time neurofeedback.

### 2.7 Image acquisition

Images were acquired with a 3 T Achieva scanner (Philips Medical Systems) using a T2*-weighted echoplanar (BOLD contrast) sequence (TR = 2000 ms, TE = 30 ms, matrix = 80 × 80, FOV = 240 mm, flip angle = 90°, voxel size 3×3×3mm; slice thickness = 3 mm, 37 slices). Before each run, five dummy volumes were collected for T1 equilibration purposes. A SENSE factor of 1.5 and dynamic stabilisation were additionally used. These parameters were based on careful sequence optimization to maximize temporal signal-to-noise[20] in brain regions that normally suffer from magnetic susceptibility effects, including the SCC and ATL. High-resolution anatomical images were acquired with a 3D turbo field echo T1-weighted sequence (TR 7.1 s, TE 3.4 s, matrix 240 × 240, FOV 240 mm, slice thickness 1 mm, 170 slices). Head motion was restricted by using foam padding and straps over the forehead and under the chin.

### 2.8 Off-line analyses

At the individual subject level, linear regression coefficients for the slope of z-transformed ATL signal time-course as the predictor of z-transformed SCC signal time-course in each condition (guilt, indignation) in the pre- and post-training acquisition as the outcome variables were derived from a general linear model in IBM SPSS 23 (http://www.ibm.com/analytics/us/en/technology/spss/) for each subject by modelling the interaction of z-transformed ATL signal time-course with two factors: condition (guilt, indignation) and time (pre-, post-training). The z-transformation was undertaken to obtain standardised regression coefficients. Cohen's d effect sizes were computed for each regression coefficient using the formula: 2 × t-value/square root of degrees of freedom (df,(Rosenthal and Rosnow, 1991)).

At the group level, these individual subject effect sizes were entered into a repeated measures ANOVA with two within-subject factors (condition: guilt, indignation, time: pre-, post-training) and one between-subject factor (group: ACTIVE, CONTROL). To further examine and estimate the confidence intervals of the observed three-way interaction between condition, time, and group, we computed the difference between the regression coefficient effects for guilt vs. indignation for post- vs. pre-training for each individual. The resulting measure was examined using an independent *t*-test to compare the two intervention groups which as expected resulted in the same *p*-value as the interaction term produced by the repeated measures ANOVA. The alpha-level was set to *p* = .05, two-tailed test. Clinical trial statisticians usually recommend using an ANCOVA of the post-intervention outcome measures with baseline as a covariate for complete data. The reason why we have chosen a different approach here for our imaging data is, because we have two related imaging outcome measures (guilt and indignation) which we sought to contrast against each other, but also look at separately. To realise this in an ANCOVA, we would have had to use a Multivariate ANCOVA with 7 predictors to model all the interactions between baseline connectivity and intervention group. With an n of 28 this would have entailed a massive overfitting problem, rendering the model probably not reproducible (Stevens, 2009).

## 3 Results

### 3.1 Primary outcome measure

As predicted, we found an increase in ATL-SCC correlation for guilt vs. indignation after training compared to before training in the ACTIVE group as compared with the CONTROL group (Table 2). Contrary to our predictions, however, we found a trendwise decrease in ATL-SCC correlation during the indignation condition after compared to before training in the ACTIVE group and an increase in the CONTROL group (Table 2). This was in contrast to our prediction of finding a difference between groups regarding ATL-SCC correlation during the guilt condition.

**Table 2|:** Intervention group comparisons on primary outcome measures.

Measure (sample size)	Pre-training				Post-training				Active vs. Control post- vs. Pre-training *t*-test					Repeated measures ANOVA							
	Active		Control		Active		Control							Condition		Time		Time × Condition		Group × Time × Condition	
	m	sd	m	sd	m	sd	m	sd	diff	se	p	95% CI	T	F	p	F	p	F	p	F	p
Guilt: ATL-SCC regression effect (n = 28)	0.14	0.55	0.15	0.64	0.12	0.43	0.18	0.46	0.05	0.30	0.88	−0.56 to.65	0.2	1.9	0.18	0.008	0.93	0.11	0.75	5.6	0.03*
Indignation: ATL-SCC regression effect (*n* = 28)	0.40	0.60	0.06	0.53	0.13	0.42	0.28	0.36	0.50	0.25	0.07	−0.04 to 1	1.9								
Guilt vs. Indignation: ATL-SCC regression effect (n = 28)	−0.26	0.38	0.09	0.40	−0.01	0.28	−0.10	0.38	−0.43	0.18	0.03*	−0.81 to −0.06	−2.4								

*Significant at *p* = .05, 2-sided. n = 14 in each group. Between-group Cohen's d scores were computed from the means and pooled standard deviations (Cohen, 1988) of the differences between Post vs. Pre-training. Mean differences and 95% confidence intervals were taken from independent samples *t*-test for differences between Post vs. Pre-training. CI = confidence interval, m = mean, sd = standard deviation, se = standard error, diff = difference of means, d = Cohen's d. For a graphical depiction of the values reported in this table, please see Supplementary Fig. 5. The ACTIVE group had a significantly lower ATL-SCC regression effect for guilt vs. indignation prior to training compared with the CONTROL group (diff = 0.35, se = 0.15, *p* = .03, 95% CI:0.045 to 0.66), and there was a weak trend towards higher regression effects for indignation in the ACTIVE vs. CONTROL group prior to training (diff = −0.34, se = 0.21, *p* = .13, 95% CI:−0.77 to 0.10) with no group differences in ATL-SCC regression effects for guilt (diff = 0.01, se = 0.23, *p* = .95, 95% CI: −0.45 to 0.48).

Further secondary analyses considering pre-training differences between groups in ATL-SCC connectivity (see Supplementary Results), confirmed our primary analysis that the ACTIVE group showed higher post-training ATL-SCC regression effects for guilt vs. indignation compared with the CONTROL group. Furthermore, this secondary analysis showed that this result was associated with the interaction of intervention group and pre-training ATL-SCC regression effects. This interaction effect was driven by a negative association of pre-training with post-training ATL-SCC regression effects for guilt vs. indignation in the ACTIVE group and a positive association in the CONTROL group. This indicated that patients in the ACTIVE group with low connectivity between the ATL and SCC for guilt vs. indignation prior to neurofeedback training were more likely to increase the levels of connectivity through training when compared with those who had high levels of connectivity prior to training already. The positive association of pre-training and post-training ATL-SCC connectivity in the CONTROL group reflected that the control intervention was aimed at stabilising pre-training levels of connectivity. Further, these secondary analyses showed that the weak trendwise neurofeedback-training effect difference between intervention groups on ATL-SCC regression coefficients for indignation in our primary data analysis disappeared when correcting for pre-training differences in ATL-SCC connectivity for indignation.

### 3.2 Planned secondary outcome measures

We had missing secondary outcome measures in some participants who did not complete all questionnaires (Table 3). As expected, the intervention did not alter Beck Depression Inventory scores in either group which was used to assess the safety of the intervention (Table 3). There were also no adverse events observed. As predicted, we found an increase in global self-esteem as measured on the Rosenberg self-esteem scale after training compared to before training in the ACTIVE group compared with the CONTROL group (Table 3). As one would expect, the level of neurofeedback-induced increases in self-esteem post- vs. pre-training correlated with the level of increases in ATL-SCC connectivity for guilt vs. indignation (*r* = 0.47, *p* = .02, *n* = 23). Contrary to our expectations, we did not find training effects for self-hate on the Interpersonal Guilt Questionnaire after training compared to before training (Table 3).

**Table 3|:** Intervention group comparisons on pre-registered secondary and exploratory outcome measures.

Measure (sample size)	Pre-training				Post-training				Active vs. Control post- vs. Pre-training *t*-test					Repeated measures ANOVA					
	Active		Control		Active		Control							Group		Time		Group × Time	
	m	sd	m	sd	m	sd	m	sd	diff	se	p	95% CI	d	F	p	F	p	F	p
BDI (PRE:28, POST:24)	4.7	4.4	8.1	8.1	4.7	4.2	7.2	7.5	0.58	1.05	0.59	−1.60 to 2.76	0.23	1.20	0.29	2.27	0.15	0.31	0.59
IGQ-Self-hate (PRE:24, POST:21)	27.4	4.9	30.9	7.4	27.9	4.8	29.9	6.9	0.43	2.17	0.79	−4.12 to 4.98	0.09	0.83	0.37	0.22	0.64	0.04	0.85
Rosenberg Self-esteem (PRE:27, POST:24)	22.1	4.0	22.4	3.8	24.2	2.9	21.8	5.7	3.34	1.44	0.03*	0.35 to 6.33	0.98^+^	0.70	0.41	0.65	0.43	5.40	0.03*
Negative Affect (PANAS) (PRE:28, POST:28)	3.8	4.0	3.9	4.6	4.1	4.2	3.5	5.1	0.64	1.52	0.68	−2.49 to 3.77	0.16	0.03	0.87	0.002	0.96	0.18	0.68
Positive Affect (PANAS) (PRE:28, POST:28)	25.0	7.9	23.1	9.2	18.6	8.7	17.9	10.7	−1.29	2.77	0.65	−6.99 to 4.42	0.18	0.16	0.69	17.39	0.0003*	0.21	0.65
Guilt vs. Indignation intensity ratings (PRE:28, POST:28)	−0.07	1.33	0.07	1.33	−0.50	0.65	0.07	1.33	−0.43	0.40	0.30	−1.26 to 0.40	0.40	0.78	0.39	1.14	0.30	1.14	0.30

*Significant at p = .05, 2-sided.Between-group Cohen's d scores were computed from the means and pooled standard deviations (Cohen, 1988) of the differences between Post vs. Pre-training. Mean differences and 95% confidence intervals were taken from independent samples t-test for differences between Post vs. Pre-training. CI = confidence interval, m = mean, sd = standard deviation, se = standard error, diff = difference of means, d = Cohen's d. ^+^After excluding one outlier in the active group (outside of ±2.5 standard deviations from the mean), the intervention's effect on self-esteem remained large: d = 0.91. The Positive and Negative Affect Schedule(Watson et al., 1988b) was used to explore affective changes of how participants felt at the moment. Ratings of guilt and indignation-evoking events were obtained prior and after the scan. In the scanner, after each run the participants were asked over the intercom to rate on a scale from 0 to 9 how much they felt guilt or indignation. Analyses of Covariance on post-intervention outcomes with pre-intervention measures as covariates confirmed the repeated measures ANOVA results (Supplementary Table 1).

### 3.3 Unplanned exploratory outcomes and confounding variables

Interestingly, both intervention groups showed a decrease in positive affect as measured on the PANAS scale after training compared to before training with no changes on negative affect (Table 3). There were no intervention effects on the rated intensity of guilt and indignation feelings during the scan (Table 3).

Potentially confounding differences between the intervention groups were examined (Table 4) and apart from a much lower average level of the thermometer scale visual feedback in the ACTIVE group during guilt neurofeedback compared with the CONTROL group, there were no differences between the intervention groups on baseline Hamilton Depression Scale, Beck Depression Inventory scores, movement parameters during scanning, and thermometer visual feedback position during indignation.

**Table 4|:** Intervention group comparisons on potentially confounding variables.

Measure (sample size)	Active		Control					
	m	sd	m	sd	diff	se	p	95% CI
Age	45.1	13.7	45.3	17.5	−0.21	5.9	0.97	−12.4 to 12.0
Years of Education	15.8	3.4	15.0	1.9	0.79	1.1	0.46	−1.4 to 2.9
Baseline Hamilton Depression Scale score (28)	2.1	1.9	1.9	2.8	0.21	0.90	0.81	−1.63 to 2.06
Baseline Rosenberg Self-esteem (27)	22.1	4.0	22.4	3.8	−0.24	1.50	0.87	−3.34 to 2.85
Baseline BDI (28)	4.7	4.4	8.1	8.1	−3.43	2.47	0.18~	−8.58 to 1.73
Baseline Global Assessment of Functioning Scale (28)	87.9	4.7	90.0	4.8	−2.14	1.8	0.24	−5.8 to 1.6
Pre-training movement parameters (28)	0.56	0.37	0.55	0.34	0.01	0.14	0.94	−0.27 to 0.29
Post-training movement parameters (28)	0.63	0.56	0.84	0.74	−0.21	0.25	0.40	−0.72 to 0.30
Average thermometer feedback position [%] for Guilt (28)	44.0	3.9	84.9	1.5	−40.98	1.11	<0.0001*^~^	−43.34 to −38.63
Average thermometer feedback position [%] for Indignation~ (28)	84.3	1.6	84.4	2.6	−0.09	0.82	0.91	−1.80 to 1.61

*=significant at p = .05, 2-sided. Mean differences and 95% confidence intervals were taken from independent samples t-test for differences between groups. CI = confidence interval, m = mean, sd = standard deviation, se = standard error, diff = difference of means, d = Cohen's d. ~Unequal variances assumed as Levene's Test significant at *p* = .05. There were also no group differences between movement parameters during the neurofeedback training runs (*t* < 0.01,*p* > .91).

Individualized ROIs showed that there was considerable individual variability regarding which subregions of the ATL and SCC ROIs exhibited the highest activation and were thus used for training (Fig. 1c&d). The anterior superior temporal gyrus was part of the individualized ROI more often than the sulcus. Within the SCC, anterior portions of the subgenual cingulate falling into Brodmann Area 24 were most commonly part of the individualized ROIs rather than the more posterior inferior subgenual cortex (Brodmann Area 25, Fig. 1e&f). The overlap between the more anterior and callosal SCC region from (Green et al., 2012) in red shows that it was more often part of the individualized ROIs than the more posterior inferior subgenual cortex region found in a subsequent paper (Lythe et al., 2015) in blue (Fig. 1e&f).

## 4 Discussion

We confirmed our more specific pre-registered hypothesis (aim 2) that compared to the CONTROL intervention, the ACTIVE neurofeedback intervention would lead to an increase in ATL-SCC connectivity for guilt relative to indignation, whilst we were unable to demonstrate that the intervention increases connectivity for guilt on its own (aim 1). We also confirmed our prediction that there would be no safety concerns by showing stable Beck Depression Inventory scores (aim 3). Intriguingly, we were able to demonstrate an effect of the intervention in increasing global self-esteem (aim 4) despite this measure's focus on general feelings about oneself rather than a specific focus on the current state. Further strengthening the assumption that this training-induced increase in self-esteem was a result of the ATL-SCC connectivity increase for guilt vs. indignation, we found a positive correlation between self-esteem and connectivity changes.

Two additional findings render a non-specific boost in positive emotions or perceived success as explanations for this effect unlikely. Firstly, the ACTIVE intervention appears to have been more difficult and thus patients had a much lower thermometer level display on average, thus getting much less positive feedback on their performance than the CONTROL intervention group. The high success rates of stabilisation neurofeedback show that this was an adequate control intervention which was unlikely to have interfered with self-efficacy perceptions shown to be important for neurofeedback outcomes (Mehler et al., 2018). Interestingly, self-esteem in the ACTIVE group increased despite lower levels of positive feedback and this did not lead to a group difference on training-induced changes in positive emotions as measured on the PANAS scale which focused on how people felt at this moment. The second finding suggesting that non-specific placebo-like effects of undergoing neurofeedback were unlikely was that both intervention groups reported lower positive emotions after training on the PANAS scale than before training which reflects qualitative reports of participants that the neurofeedback training was quite exhausting. This also shows that although habituation and fatigue could have affected connectivity changes in both groups, they were unlikely contributors to group differences.

Our finding of a direct relationship between ATL-SCC connectivity and global self-esteem in MDD provides further evidence for the important role of this neural system for self-worth in MDD (Green et al., 2012). This direct link between self-blaming biases and self-worth in MDD provides further evidence for the revised learned helplessness model of MDD vulnerability (Abramson et al., 1978). Our findings are also in keeping with the hypothesis that self-blame-selective functional disconnection of right superior ATL representations of differentiated conceptual meaning of social behaviour (Pobric et al., 2016; Zahn et al., 2009a; 2007; 2009b) makes individuals more vulnerable to attribute self-blame in an overgeneral way (Green et al., 2012; 2010).

A more recent study (Lythe et al., 2015), whose results were not available when designing the current trial, however, complicates this ATL-SCC disconnection hypothesis. This is because unexpectedly, higher rather than lower ATL-subgenual connectivity for self- vs. other-blame predicted risk of subsequent recurrence in remitted MDD. An anatomically more precise examination of these findings (Price and Drevets, 2010), however, may offer clues as to resolving these discrepancies. Self-blame-selective decreases in connectivity were found close to the corpus callosum (Green et al., 2012) (MNI: x-6, y = 22,z = 0) in a region between the more anterior BA24 which is close to the corpus callosum and the more posterior inferior subgenual cortex, BA25, which is not adjacent to the corpus callosum. In contrast, self-blame-selective increases in connectivity were found very clearly in the posterior inferior subgenual region ((Lythe et al., 2015), MNI x = 2,y = 14,z = −6), which corresponds to the core of BA25 (Price and Drevets, 2010) and were not adjacent to the corpus callosum. Our more detailed anatomical characterisation of individualized SCC ROIs for the current study showed that it was indeed more frequently the more anterior subgenual cingulate area (BA24) which was used for training, whereas there were only 4 participants with ROI voxels falling into the more posterior inferior area (BA25 (Price and Drevets, 2010), Fig. 1f). This is in keeping with the hypothesis that the anterior SCC (BA24) and the posterior inferior subgenual cortex (BA25) play distinct roles in self-blaming biases and MDD (Fu et al., 2013) which will need to be investigated in future studies.

Our finding that patients with lower baseline levels of ATL-SCC connectivity for guilt relative to indignation had stronger training effects in the ACTIVE group should be used to design future trials by stratifying patients on the basis of their functional connectivity patterns when assigning them to a blame rebalance neurofeedback intervention. This is likely to increase the treatment effects and thus the statistical power of the trial. It remains unclear why the ACTIVE group had lower baseline connectivity for guilt vs. indignation compared with the CONTROL group, but one clue is offered by one participant in the control group with the highest baseline connectivity levels in the study, who showed potential bipolar features. Future trials should employ DSM5 criteria with its improved sensitivity for bipolar spectrum conditions which we may have under-detected in the current DSM-IV-based study, leading to potentially uncontrolled group differences in the frequency of patients whose condition would have been better captured by a bipolar spectrum diagnosis.

### 4.1 Limitations

Potential limitations of our study were that we cannot determine how representative our sample was of MDD patients seen in general outpatient specialist settings as we did not collect data on how many patients were pre-screened for eligibility by referring psychiatrists. Another limitation was that this study was designed as a proof-of-concept study with a limited sample size, so that our results need to be replicated in a larger sample, ideally with repeated neurofeedback sessions, and a longer follow-up period to demonstrate clinical benefits and further safety data.

## 5 Conclusions

Patients with MDD were shown to be able to modulate guilt-selective ATL-SCC functional connectivity after a single neurofeedback session and this improved their self-esteem. Furthermore, increases in self-esteem correlated with connectivity increases. Non-specific effects such as perceived success cannot explain these findings. Whilst preliminary, this is in keeping with the hypothesis of a relevant direct or indirect relationship between ATL-SCC connectivity and self-esteem, although confounding differences between the groups other than the received ATL-SCC connectivity feedback cannot be ruled out (Mehler and Kording, 2018), and provides the clinical proof-of-concept for a novel functional MRI treatment target in MDD. Further studies are needed to investigate the differential roles of more posterior inferior and more anterior superior sectors of the subgenual region in the pathophysiology of MDD. This will be important in order to optimise future neurofeedback treatment protocols and for understanding the pathophysiology of self-blaming biases in MDD. Our study contributes to the emerging evidence on novel neurofeedback treatment targets for psychiatric disorders (Sitaram et al., 2017).

## Declaration of Competing Interest

RZ: Lundbeck-sponsored presentation on neurofeedback, co-investigator Livanova-funded study; Industry advice via Guidepoint Global; Industry collaborations with EMIS PLC and Alloc Modulo LTD. Private Clinical Practice at The London Depression Institute. AHY: Paid lectures and advisory boards for the following companies with drugs used in affective and related disorders: Astrazenaca, Eli Lilly, Lundbeck, Sunovion, Servier, Livanova, Janssen; Consultant to Johnson & Johnson; Lead Investigator for Embolden Study (AZ), BCI Neuroplasticity study and Aripiprazole Mania Study; Investigator initiated studies from AZ, Eli Lilly, Lundbeck, Wyeth, Janssen. JM: Shareholder of Rede D'Or hospitals (Brazil) and a managing partner of VHM LLC. (US). All these interests are unrelated to the study. The other authors have no conflicts of interest to declare.

## Appendix A Supplementary data

Supplementary material Image 1

Consort checklist Image 2
