# Supplementary material for: Blame-rebalance fMRI neurofeedback in major depressive disorder: A randomised proof-of-concept trial
Source: Neuroimage Clin. 2019 Aug 25;24:101992. doi: 10.1016/j.nicl.2019.101992 (PMC6737344; doi:10.1016/j.nicl.2019.101992)
Supplement: Supplementary file 1 — Supplementary material [file mmc1.docx]

**SUPPLEMENTARY INFORMATION**

**Blame-rebalance fMRI neurofeedback in major depressive disorder:**

**A randomised proof-of-concept trial**

Roland Zahn^1,5^, Julie H. Weingartner^1^, Rodrigo Basilio^1^, Patricia Bado^1,2^, Paulo Mattos^1^, João R. Sato^1,3^, Ricardo de Oliveira-Souza^1,4^, Leo F. Fontenelle^1^, Allan H. Young^5^,

Jorge Moll^1*^

*^1^ Cognitive and Behavioral Neuroscience Unit and Neuroinformatics Workgroup, D’Or Institute for Research and Education (IDOR) – Rio de Janeiro, Brazil*

*^2^ Instituto de Ciências Biomédicas (ICB), Universidade Federal do Rio de Janeiro – Rio de Janeiro, Brazil*

*^3^ Center for Mathematics, Computation, and Cognition, Universidade Federal do ABC – Santo André, Brazil*

*^4^ Gaffrée e Guinle University Hospital, Federal University of the State of Rio de Janeiro – Rio de Janeiro, Brazil*

*^5^ Centre for Affective Disorders, Institute of Psychiatry, Psychology & Neuroscience, King's College London, United Kingdom*

*Corresponding author

Jorge Moll – *e*-mail: jorge.moll@idor.org

Cognitive and Behavioral Neuroscience Unit

D’Or Institute for Research and Education

Rua Diniz Cordeiro, 30/3° andar

Rio de Janeiro–22281-100, Brazil

Phone: [+55 21 3883-6000](tel:%2B55%2021%202538%203541)

*e*-mail: jorge.moll@idor.org

**Supplementary Methods**

*Full instructions given to neurofeedback participants*

Participants were given the following instructions in written and oral form (English translation): “On the screen you will see a bar filled with colour that can reach different levels. The levels are calculated from your brain activity patterns with a delay of 6 seconds. Whilst in the scanner, keep thinking about the situation related to the cue word that will be displayed (guilt in the guilt condition, indignation in the indignation condition).  Try to bring up the level to which the bar is filled with colour to the top. If you get to the top, try to keep it there. If the level drops, try to bring it up again.”

*Defining the range in moving target neurofeedback algorithm during real-time fMRI*

The thermometer-like visual feedback display was created by defining the maximum and minimum of the range of displayed correlations in the following way:

- Maximum = average Pearson correlation coefficient for sliding time window over previous 10 volumes weighted with a sigmoid function (see Supplementary Figure 1) to give more weight to more recent volumes) + 1 standard deviation (SD)
- Minimum = average Pearson correlation coefficient for sliding time window over previous 10 volumes (weighted by sigmoid function) – 1 SD.


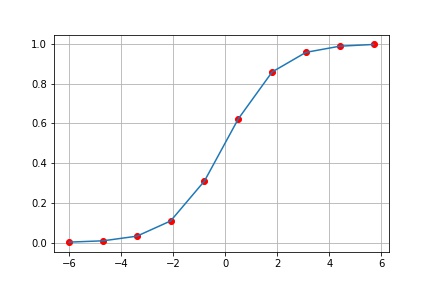


**Supplemental Figure 1|** The sigmoid values were calculated sampling 10 values ranging from -6 to 6 and calculating the sigmoid function as y = 1 / (1+exp(-x)). To clarify, if c^i^ was the i^th^ sigmoid value, the weight w^i^ was equal to c^i^ divided by the sum of all ten c^i^ values.

*Movement parameters during real-time fMRI*

Root mean squares (RMS) of movement parameters for translation and rotation were tracked on an ongoing basis and the neurofeedback screen displayed a warning to participants and investigators in real-time if movement exceeded allowable levels. Excessive movement was assumed if volumes showed movement parameters outside the minimum and maximum RMS range; these were discarded from computing neurofeedback signals The maximum RMS of the acceptable range was defined as the average RMS of the previous 40 volumes (weighted by sigmoid function) + 0.4 and the minimum RMS of the acceptable range as the average RMS of the previous 40 volumes (weighted by sigmoid function) – 0.4. All volumes outside this movement range were not considered for the correlation curve. This is because the maximum (one direction) and minimum (opposite direction) reflect deviations from a stable state rather than more or less movement.

**Supplementary Results**

In order to determine whether our results could be reproduced when correcting for pre-training differences between groups in ATL-SCC connectivity, we conducted a general linear model probing the main effects and interactions of pre-training ATL-SCC connectivity and intervention group on post-training ATL-SCC connectivity. We found that only when examining guilt vs. indignation connectivity, there was an interaction between intervention group and pre-training ATL-SCC connectivity (F[24,3]=4.25, p=.05) with no main effects (F<.23, p>.64). There were no main effects or interactions when running this model for ATL-SCC regression effects for guilt or indignation as separate outcome variables respectively (F<1.1, p>.31).

To provide a description of the spatial distribution of correlations with individualised right ATL ROIs, we displayed the consistency of at least moderate positive (Supplementary Figure 3) and negative (Supplementary Figure 4) correlations (Pearson’s r of at least .30) in each voxel of the brain. We refrained from inferential analyses at the whole brain level for several reasons: 1) FRIEND software and FSL, on which it is based, have not incorporated tools to compare simple correlations between conditions and groups at a voxel-based level, 2) Other software for offline fMRI analyses, such as Statistical Parametric Mapping (SPM) uses a more complex approach to functional connectivity analyses as a difference in regression slopes between conditions after covarying baseline connectivity and activation in each condition (Friston et al., 1997), which would make comparison with the simplified real-time analysis carried out by FRIEND difficult, 3) Given our sample size and the two factors, we were interested in (Intervention group and Condition), our study was probably heavily under-powered for multiple comparison correction across the whole brain which is why we did not include such an analysis in our registered trial protocol.

Our descriptive analysis showed that right superior ATL correlations were positive overall (Supplementary Figure 3) with no consistent negative correlations (Supplementary Figure 4). The most consistent positive correlations (voxel overlap in at least 6 participants) were found within ipsilateral and contralateral anterior and posterior temporo-occipital, thalamus and basal ganglia, amygdala, as well as dorso- and ventromedial frontal cortex including the subgenual area.

**Supplementary Table 1|** Intervention group comparisons on pre-registered secondary and exploratory outcome measures: ANCOVA

|  | Group | |  |
| --- | --- | --- | --- |
| MEASURE (sample size) | F | p | |
| BDI  (PRE:14, POST:12) | .004 | .95 | |
| IGQ-Self-hate  (PRE:24, POST:21) | .04 | .84 | |
| Rosenberg Self-esteem  (PRE:27, POST:24) | 5.32 | .03 | |
| Negative Affect (PANAS)  (PRE:28, POST:28) | .18 | .67 | |
| Positive Affect (PANAS)  (PRE:28, POST:28) | .10 | .76 | |
| Guilt vs. Indignation  intensity ratings  (PRE:28, POST:28) | 2.51 | .13 | |

*=significant at p=.05, 2-sided. CI=confidence interval, m=mean, sd=standard deviation, se=standard error, diff=difference of means. The Positive and Negative Affect Schedule(Watson et al., 1988) was used to explore affective changes of how participants felt at the moment. Ratings of guilt and indignation-evoking events were obtained prior and after the scan. In the scanner, after each run the participants were asked over the intercom to rate on a scale from 0 to 9 how much they felt guilt or indignation. Analyses of Covariance (ANCOVA) on post-intervention outcomes with pre-intervention measures as covariates and intervention group as predictor were carried out to confirm the repeated measures ANOVA results (Table 3).

**
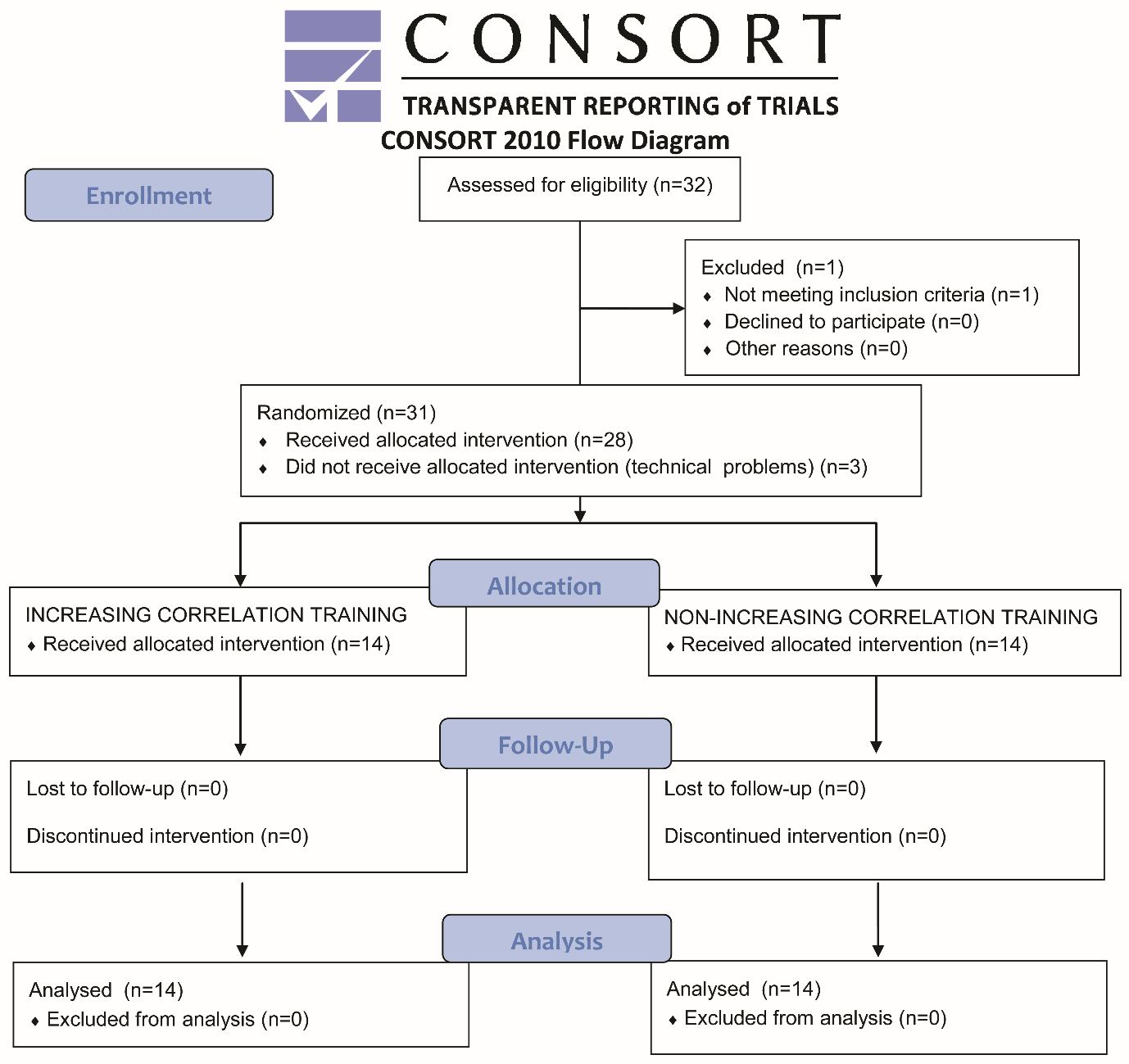
Supplementary Figure 2**| The CONSORT flow chart diagram shows that most screened patients were included. This is due to the fact that patients were referred from psychiatrists familiar with the inclusion/exclusion criteria. We have not asked referring psychiatrists to formally undertake an eligibility screening of their caseload.


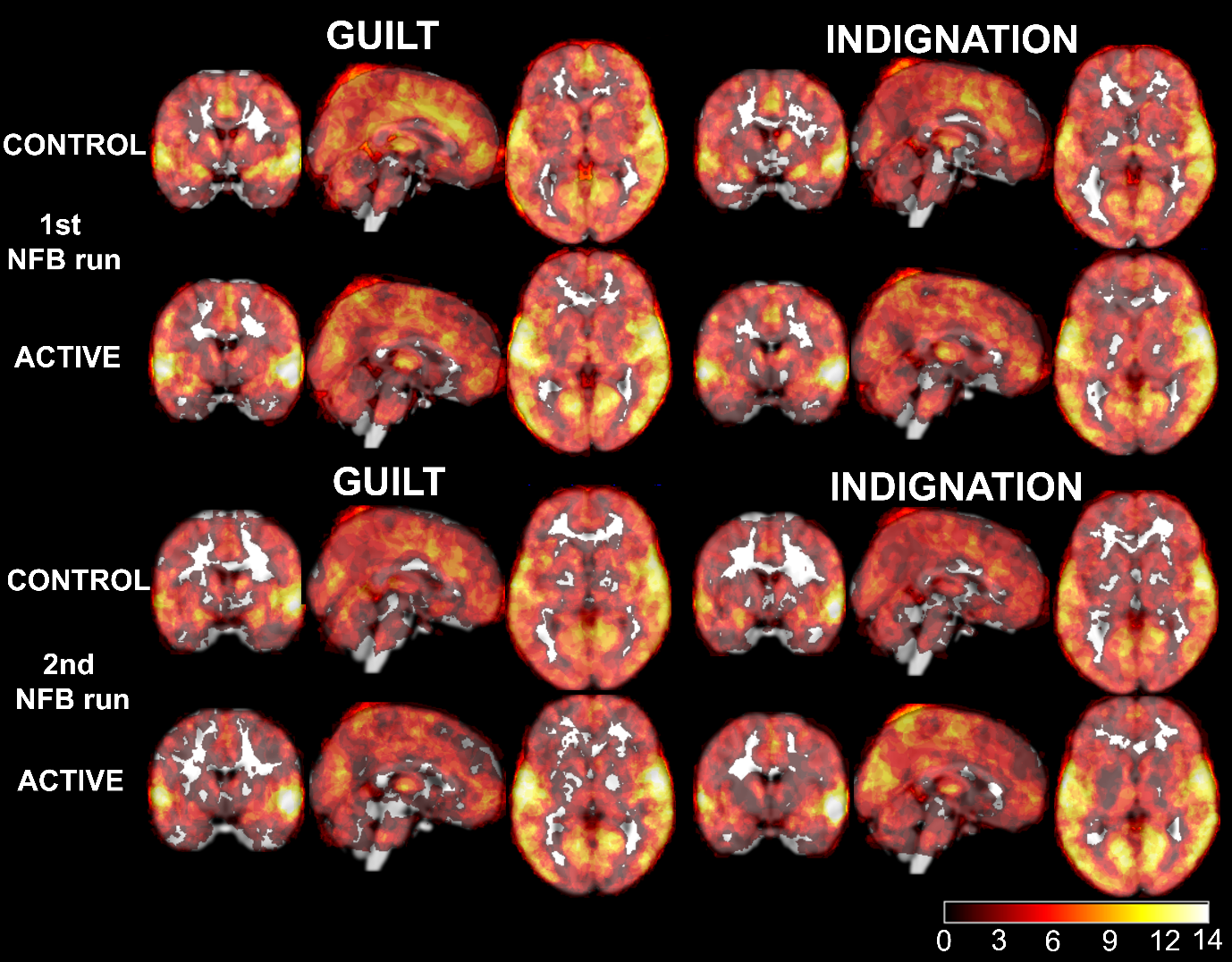


**Supplementary Figure 3**| For exploratory purposes, interindividual consistency is displayed as the colour-coded number of participants (n=0-14) showing positive correlations (Pearson’s correlation coefficients ≥ .3) between individualised right superior anterior temporal lobe (ATL) regions of interest with each voxel of the brain. Correlations were obtained from FRIEND software as averages for the 1^st^ and 2^nd^ neurofeedback (NFB) runs in the ACTIVE (n=14) and CONTROL (n=14) neurofeedback groups for guilt and indignation conditions respectively and individual maps were transformed into standard space using the FSL-based routines implemented in FRIEND. Maps were projected onto coronal, sagittal, and axial slices through the origin (MNI x=0, y=0, z=0) of the MNI152_T1_1mm_brain.nii template using MRIcron(Rorden and Brett, 2000).

**
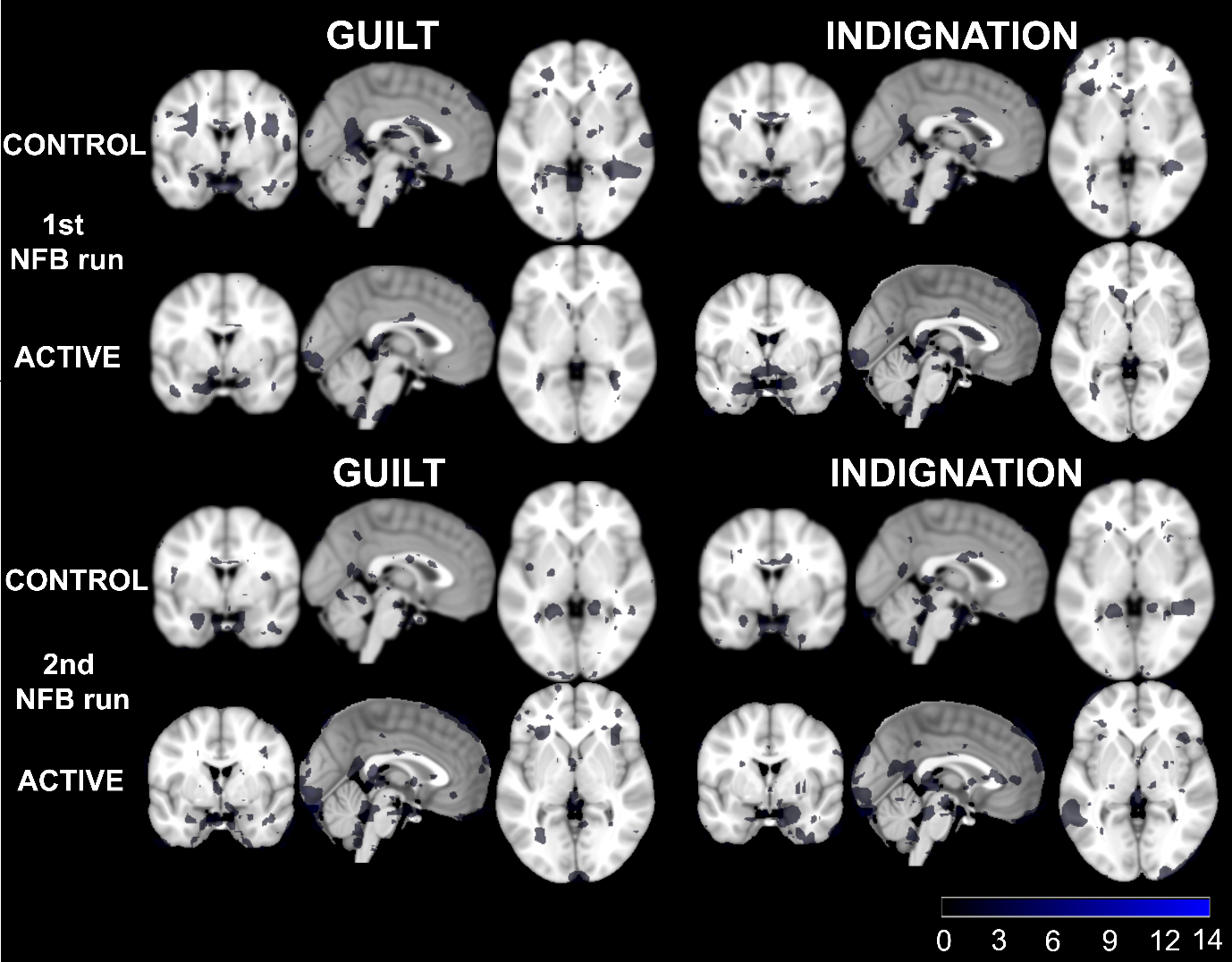
Supplementary Figure 4**| For exploratory purposes, interindividual consistency is displayed as the colour-coded number of participants (n=0-14) showing negative correlations (Pearson’s correlation coefficients ≤-.30) between individualised right superior anterior temporal lobe (ATL) regions of interest with each voxel of the brain. Correlations were obtained from FRIEND software as averages for the 1^st^ and 2^nd^ neurofeedback (NFB) runs in the ACTIVE (n=14) and CONTROL (n=14) neurofeedback groups for guilt and indignation conditions respectively and individual maps were transformed into standard space using the FSL-based routines implemented in FRIEND. Maps were projected onto coronal, sagittal, and axial slices through the origin (MNI x=0, y=0, z=0) of the MNI152_T1_1mm_brain.nii template using MRIcron(Rorden and Brett, 2000).

**
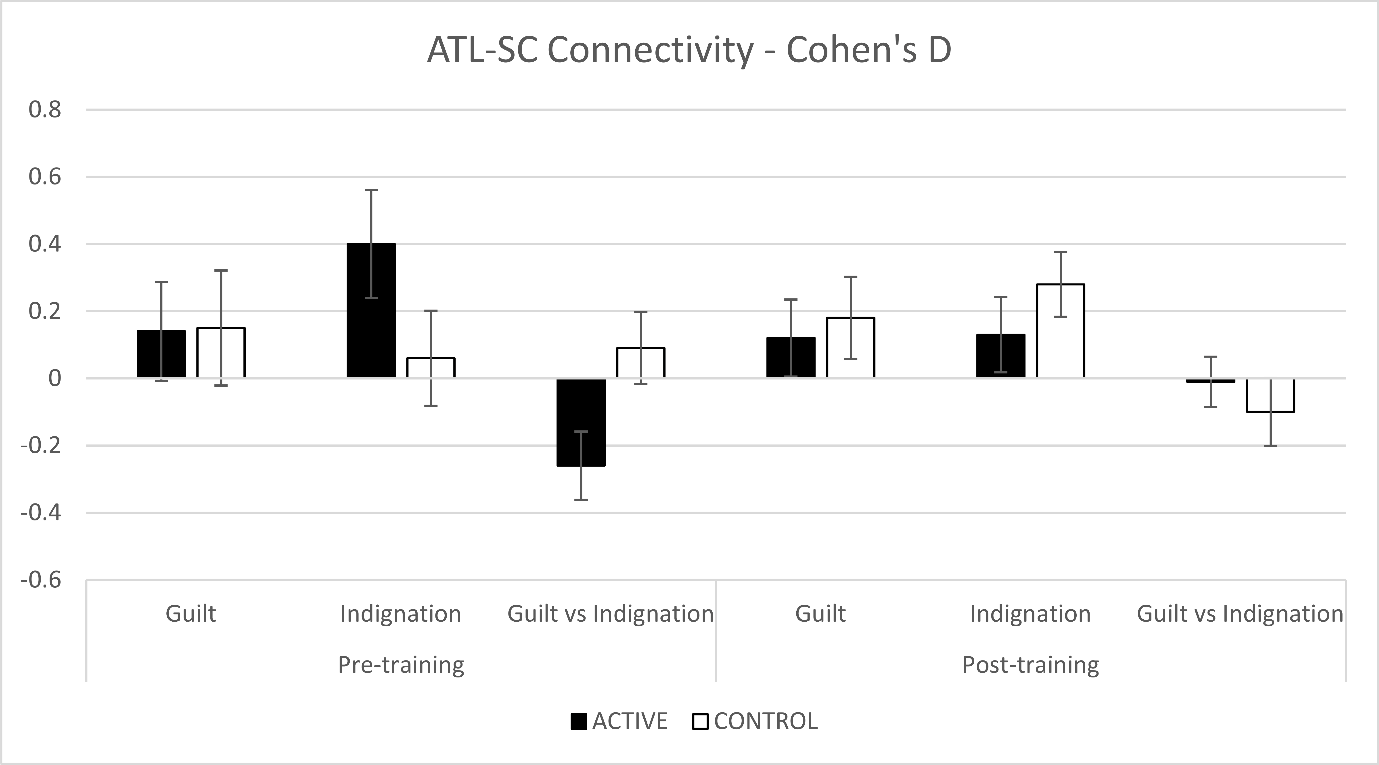
**

**Supplementary Figure 5**| Cohen’s d value means and standard errors for the regression coefficients representing connectivity between signal in the individualised right superior anterior temporal lobe (ATL) and subgenual cingulate (SC) regions of interest. Signal time courses were obtained from FRIEND software for the pre- and post-neurofeedback (NFB) runs in the ACTIVE (n=14) and CONTROL (n=14) neurofeedback groups for guilt and indignation conditions respectively. Please see Table 2 for the corresponding means and standard deviations.

**
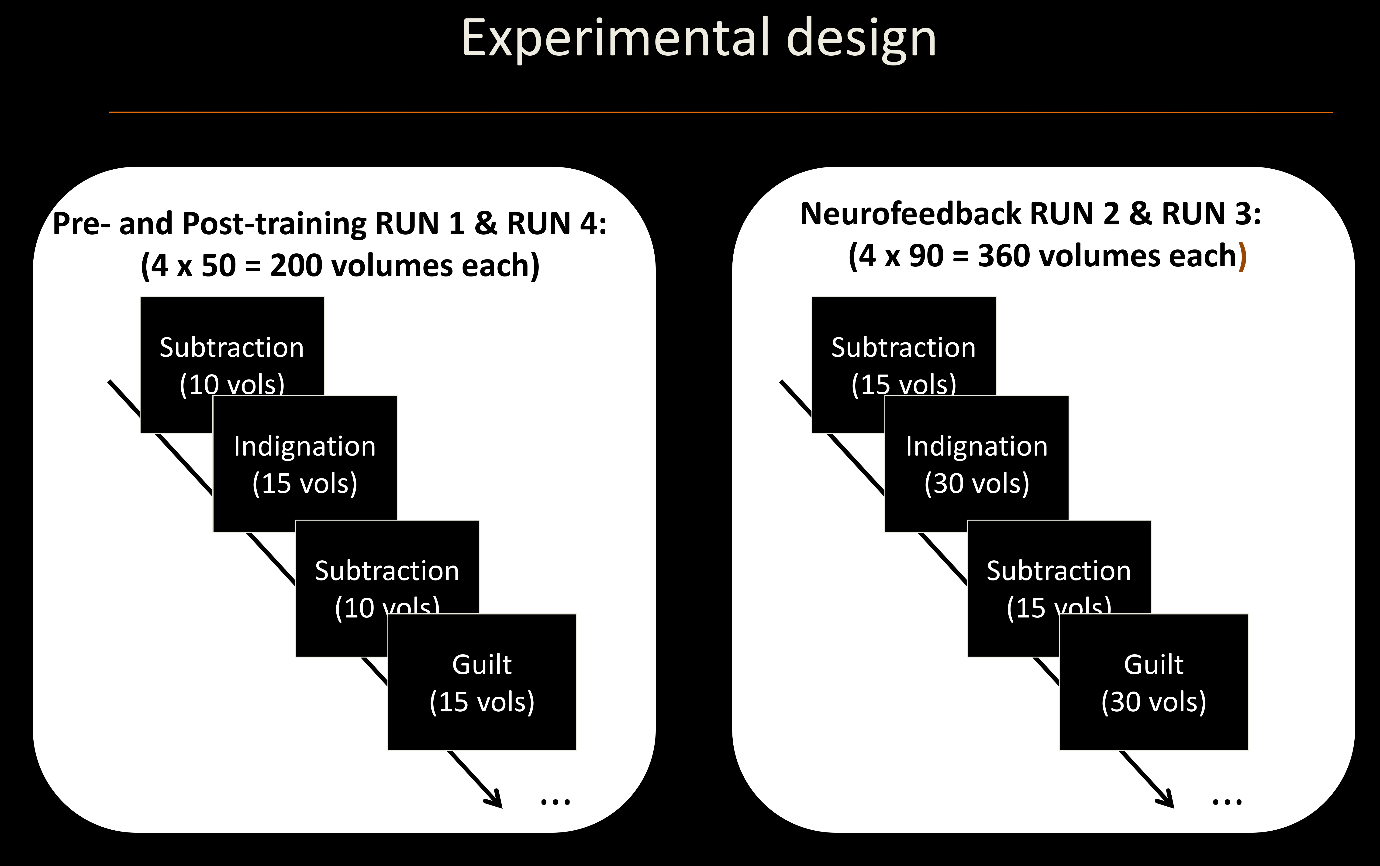
**

**Supplementary Figure 6**| The experimental design of the four fMRI runs is summarised. Each run consisted of a sequence of four units consisting of four blocks as depicted. In the neurofeedback runs, during the indignation condition, visual feedback reinforced stabilisation of the preceding degree of correlation between the ATL and SCC in both intervention groups. The two intervention groups were: *ACTIVE: GUILT-INCREASE-CORRELATION:* Visual feedback reinforced increasing the correlation in fMRI signal between the right superior ATL and SCC regions during retrieval of guilt-related events. *CONTROL: GUILT-STABILISE-CORRELATION:* Visual feedback reinforced stabilisation of the preceding degree of correlation in fMRI signal between the right superior ATL and SCC regions during the retrieval of guilt-related events.

**
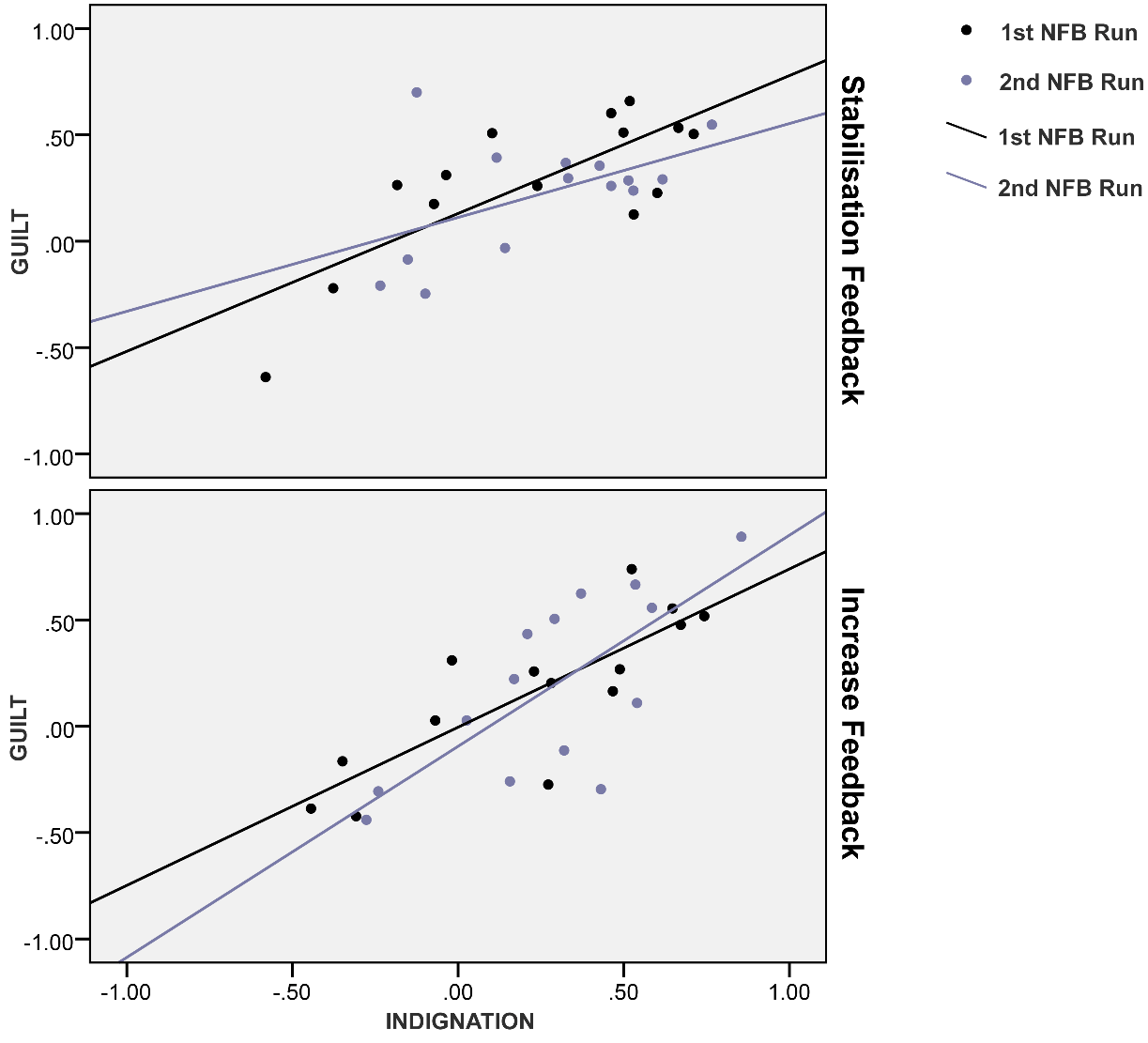
**

**Supplementary Figure 7**| This exploratory scatter plot displays Pearson’s r-values for signal time series in each condition (guilt and indignation) and neurofeedback (NFB) run between the individualised right superior anterior temporal and subgenual cingulate regions of interest for each participant in the CONTROL and ACTIVE intervention group. Lines represent linear regression lines depicting the relationship between Guilt and Indignation correlations across participants. One can observe the high individual variability in correlation values.

**Supplementary References**

Friston, K., Buechel, C., Fink, G., Morris, J., Rolls, E., Dolan, R., 1997. Psychophysiological and modulatory interactions in neuroimaging. Neuroimage 6, 218-229.

Rorden, C., Brett, M., 2000. Stereotaxic display of brain lesions. Behavioural Neurology 12, 191-200.

Watson, D., Clark, L.A., Tellegen, A., 1988. Development and validation of brief measures of positive and negative affect: the PANAS scales. Journal of Personality and Social Psychology 54, 1063-1070.
